# Tissue Responses to Postoperative Laser Therapy in Diabetic Rats Submitted to Excisional Wounds

**DOI:** 10.1371/journal.pone.0122042

**Published:** 2015-04-24

**Authors:** Cristiano de Loura Santana, Daniela de Fátima Teixeira Silva, Alessandro Melo Deana, Renato Araujo Prates, Amanda Pires Souza, Mariana Teixeira Gomes, Brunna Pileggi de Azevedo Sampaio, Josiane Ferraretto Shibuya, Sandra Kalil Bussadori, Raquel Agnelli Mesquita-Ferrari, Kristianne Porta Santos Fernandes, Cristiane Miranda França

**Affiliations:** Postgraduate Program in Biophotonics Applied to Health Sciences, University Nove de Julho (UNINOVE), São Paulo, São Paulo, Brazil; Cedars-Sinai Medical Center; UCLA School of Medicine, UNITED STATES

## Abstract

In a previous study about low-level laser therapy biomodulation on a full-thickness burn model we showed that single and fractionated dose regimens increased wound healing and leukocyte influx similarly when compared with untreated control. In order to verify if this finding would be similar in an impaired wound model, we investigated the effect of single and multiple irradiations on wound closure rate, type of inflammatory infiltrate, myofibroblasts, collagen deposition, and optical retardation of collagen in diabetic rats. Female Wistar rats in the same estrous cycle had diabetes induced with streptozotocin and an 8-mm excisional wound performed with a punch. The experimental groups were: control group – untreated ulcer; single-dose group – ulcer submitted to single dose of diode laser therapy (λ = 660 ± 2 nm; P = 30 mW; energy density: 4 J/cm^2^) and fractionated-dose group – ulcer submitted to 1 J/cm^2^ laser therapy on Days 1, 3, 8, and 10. The ulcers were photographed on the experimental days and after euthanasia tissue samples were routinely processed for histological and immunohistochemistry analyses. Independently of the energy density, laser therapy accelerated wound closure by approximately 40% in the first three days in comparison to the control group. Laser therapy increased acute inflammatory infiltrate until Day 3. Both laser groups exhibited more myofibroblasts and better collagen organization than the control group. The findings demonstrate that low-level laser therapy in the immediate postoperative period can enhance the tissue repair process in a diabetes model. Similar effects were achieved with laser therapy applied a single time with an energy density of 4 J/cm^2^ and applied four times with an energy density of 1 J/cm^2^. The application of laser therapy in the inflammatory phase was the most important factor to the enhancement of the tissue repair process.

## Introduction

Diabetes mellitus (DM) is a common disease with the estimated prevalence of more than 371 million people worldwide and an increasing incidence in every country. Patients with diabetes often have surgical needs due to health disorders caused mostly by chronic hyperglycemia and are at risk for postoperative complications related to the non-healing of surgical wounds [1]. DM impairs wound healing due to an imbalance in the inflammatory response, the altered production of cytokines, altered collagen synthesis, reduced angiogenesis, and reduced tensile strength [2–4]. This leads to a decrease in wound strength, poor wound contraction, an increased incidence of infection, and dehiscence, which prolong hospitalization and increase the mortality rate [5]. The World Health Organization (WHO) estimates that there will be 366 million individuals with diabetes in 191 countries by the year of 2030 [6, 7].

Low-level laser therapy has been used in the clinical setting as a complementary tool for pain relief as well as due to its anti-inflammatory effects and has also been employed to accelerate the healing process in cases of muscle injury [8, 9], burns [10], surgical wounds [11, 12] and chronic ulcers [13, 14]. The biomodulatory effects of laser therapy are based on the theory that photon energy is absorbed by cellular photoacceptor molecules, such as oxyhemoglobin, hemoglobin, cytochrome c oxidase and melanin. Once the photon energy is absorbed, the photoacceptor assumes an electronically excited state and this energy is converted into chemical energy within the cell [15]. Cytochrome c oxidase receives photons and promotes a change in the mitochondrial redox state and/or pumping of ions across the inner mitochondrial membrane as well as an increase in ATP synthesis [15]. There is also an increase in intracellular calcium (Ca^2+^), which stimulates DNA and RNA synthesis, thereby activating a cascade of intracellular signals [16]. This ultimately stimulates DNA duplication, increases protein synthesis, regulates oxidative stress, and modulates the production of different cytokines [17, 18]. These events lead to the biomodulation of different cell types involved in tissue regeneration [19], including an increase in fibroblast mitosis [20], greater angiogenesis [21, 22], changes in the synthesis of cytokines [23–25], and assistance in the conversion of fibroblasts into myofibroblasts [26].

These effects have been demonstrated in both pre-clinical studies and clinical trials [8–14, 27]. However, the lack of standardization regarding dosimetry and light delivery regimens as well as the incomplete understanding of the associated cellular and molecular mechanisms of action limit the use of this treatment modality [28]. The importance of dose versus irradiation moment is still a matter of investigation. If a single laser exposure would be enough to produce the same effect as three or four exposures, regarding the compliance of the therapy, and also the costs involved, a single application would be better [10].

The hypothesis of this study was whether lasertherapy delivery regimen would impact on the final repair tissue under hyperglycemic conditions. Considering the diabetes epidemics and that these individuals suffer traumatic injuries and surgeries, to apply lasertherapy on alternate days for more than two weeks is not a reliable therapy due to the general lack of patients’ compliance. We searched for a photobiomodulation alternative regimen that could trigger the expected tissue responses of improved healing in less time. Thus, we compared the effect of two laser delivery regimens (single dose and fractionated dose) on the postoperative repair of diabetic wounds using objective parameters of tissue healing: wound closure rate, inflammatory infiltrate type, myofibroblasts count, collagen deposition, and optical retardation of collagen.

## Materials and Methods

### Animals

Ninety female adult Wistar rats (body mass: 250 ± 50 g) were kept in vivarium in plastic cages with five animals each, with free access to water and chow, 12-h light/dark cycle, 22°C, and 70% umidity). The animals were monitored daily. This study received approval from the Animal Research Ethics Committee of University Nove de Julho (Brazil, process number: ANS 026/12) and was carried out in compliance with Brazilian ethical principles for animal experimentation.

### Chemical induction of diabetes

After fasting for 12 h with free access to water, diabetes was induced in all animals with an intraperitoneal injection of streptozotocin (Sigma-Aldrich, St. Louis, MO, USA) dissolved in 0.05 M of citrate buffer (dose: 60 mg/kg of body mass). Blood glucose levels were measured on a weekly basis. Animals with fasting blood glucose greater than 220 mg/dL and stable body mass after one week were selected for the experiment.

We used a 60 mg/kg dose to avoid unnecessary suffering and death due a 100mg/kg dose, which is highly toxic to the animals. Considering that blood glucose peak is before the 50^th^ day [29], and that we wanted to study the wound healing in the maximum hyperglycemia, we calculate the experiment to start 15 days after the diagnosis of diabetes and the last group wound end on day 21. Our experiment reproduced how lasertherapy could aid a post surgical wound in a diabetes type 1 individual, not a chronic wound in an old person.

### Injury model

Anesthesia was performed with 80 mg/kg of ketamine HCl (Dopalen, Vetbrands, SP, Brazil) and 10 mg/kg of xylazine (Anasedan, Vetbrands, SP, Brazil). Fur was removed from the back of each animal with an electric shaver and a hair removal cream (Veet Cream, SP, Brazil). The skin was then cleaned with a 0.12% chlorhexidine solution. An 8-mm surgical punch (Richter, SP, Brazil) was used to produce round wounds in the central portion of the dorsum. The animals were maintained on a warm plate (37°C) to prevent hypothermia until complete recovery from the anesthesia. Then, to prevent pain the animals received an intramuscular injection of tramadol hydrochloride (5 mg/kg) twice a day for two days.

### Experimental groups

The animals were divided into three groups with thirty animals each (Table 1).

**Table 1:** Experimental groups and treatment parameters.

Number of animals	30	30	30
**Group**	Control group (CG)	Fractionated-dose group (FDG)	Single-dose group (SDG)
**Condition**	Untreated	Laser therapy	Laser therapy
**Laser energy density**		1 J/cm^2^	4 J/cm^2^
**Laser exposition time**		26 s	104 s
**Treatment frequency**		Four times	Once

### Laser system

A gallium-aluminum-arsenide diode laser (MMOptics, São Carlos, SP, Brazil) (wavelength [λ]: 660 ± 2 nm) was employed with a beam spot of 0.04 cm^2^, which was enlarged to 10 mm in diameter using a diverging lens to ensure complete coverage of the ulcer. The output power was 30 mW. The energy density and exposure time in the single-dose and fractionated dose groups (SDG and FDG, respectively) are displayed in Table 1. The output power was measured before and after irradiation to guarantee the parameters used (LaserCheck, Coherent, Santa Clara, CA, USA).

### Wound closure rate

The animals were anesthetized as described above and placed in the prone position. Pictures were taken of the ulcers using a Canon T1i with a 100-mm Canon macro lens (Kunisaki, Oita Prefecture, Japan). The ulcers were photographed daily until the closing of the wound (i.e., 22^nd^ day of the study). The ulcerated area was measured manually with the aid of the ImageJ 1.45 program (free software, NIH, Bethesda, Maryland, USA). Measurements were compared with a fully automated numerical method for the validation of the results. A complete description of the method can be found in a paper by Deana (2013) [30]. Photos from Day 1 and the time of euthanasia were compared to determine the wound closure rate.

### Analysis of the healing morphogy, inflammatory infiltrate and myofibroblasts count

Five animals from each group were euthanized on Days 1 (2 hours after injury and laser irradiation), 3, 8, 10, 15 and 22 with an overdose of anesthesia. The ulcerated tissue was removed, fixed in 10% buffered formalin (pH 7.4) and embedded in paraffin. Three 5-μm section from each animal sample was stained with hematoxylin and eosin for morphological analysis. An experienced pathologist blinded to the allocation of the samples to the different groups performed the analysis, searched the complete extension of each sample with an light microscope (Leica Microsystems, Wetzlar, Germany) and recorded the presence/absence of ulcer, epithelization, granulation tissue, and fibrosis.

The inflammatory cells neutrophil and T lymphocyte identification was made with immunohistochemistry (described below) and it was scored as: 0 = absent, 1 = low (up to 25% of cells), 2 = moderate (25 to 50% of cells), and 3 = high (50% to 100% of cells). To do this score, the complete area of the injured tissue of all animals was analyzed in triplicate. The highest scores were recorded.

Immunohistochemical analysis was performed for the myofibroblast count and inflammatory cells identification. Serial sections of paraffin-embedded tissues (3 μm) were placed on glass slides coated with 2% 3-aminopropyltriethylsilane (Sigma-Aldrich, St. Louis, MO) and deparaffinized in xylene, followed by immersion in alcohol and incubation with 3% hydrogen peroxide diluted in Tris-buffered saline (TBS) (pH 7.4). The sections were blocked by incubation with 3% normal goat serum for 20 minutes and immersed in citrate buffer (pH 6.0) at 95°C for 20 minutes for antigen retrieval. The slides were then incubated with anti-alpha smooth muscle actin (ABCAM, ab5694), anti-neutrophil elastase (ABCAM cat 68672, 1:3500), and anti-CD3 T lymphocyte marker (- ABCAM 5690, 1:300). The samples were kept overnight at 4°C in a humidified chamber, followed by washing of the sections with TBS, incubation with N-Histofine Simple Stain (Nichirei Biosciences Inc., Tokyo, Japan) for 30 minutes and incubation in 3,3’-diaminobenzidine in a chromogen solution (Dako) at room temperature for two to five minutes. The sections were then stained with Mayer’s hematoxylin and covered. For the negative controls, the primary antibodies were replaced with 1% PBS/bovine serum albumin and non-immune mouse serum (X501-1, Dako).

To count the myofibroblasts, five consecutive microscope fields (magnification: 400 x) with the most myofibroblasts (hot spot) were photographed (Leica Microsystems, Wetzlar, Germany). An experienced pathologist blinded to the allocation of the samples to the different groups performed the analysis of the images with the aid of the ImageJ 1.45 program (free software, NIH, Bethesda, Maryland, USA), using the “cell counter” plug-in. Analysis were made in triplicate.

### Collagen deposition

The analysis of collagen deposition was performed using interference colors, which are directly proportional to the thickness and packing state of the fibers. The constant thickness of the cuts allowed the study of the packing state: wider, packed fibers appeared as orange to red and thin, less-packed fibers appeared as green [31]. For this analysis, histological cuts measuring 8 μm were obtained, stained with Picrosirius Red, and examined under a polarizing microscope (Pol-Interferencial Photomicroscope, Model 61282, Carl Zeiss, Germany). The photographs were digitalized and examined using the ImageJ 1.45 program for the quantification of each color (green, orange and red) and determination of the proportion of the different packing states.

### Optical retardation of collagen

For the quantification of the optical retardation of collagen, birefringence was measured in deparaffinized, unstained histological cuts measuring 8 μm. Readings were performed with the samples soaked in distilled water using a polarizing microscope (Pol-Interferencial Photomicroscope, Model 61282, Carl Zeiss, Germany) with a high-pressure mercury bulb (HBO 200W) for illumination and an interference filter (PIL 546) for the determination of monochromatic light at λ = 546 nm. The readings were conducted with a compensator, which introduces optical retardation of λ/4. When the difference in the optical path of the sample is equal to the retardation of the compensator, destructive interference occurs between the ordinary and extraordinary rays emanating from the sample, characterized by a dark background seen in the ocular of the microscope. The field varies in position (degrees) in relation to the light beam. Upon encountering this dark field, the angle in the microscope is read by the accessory that complements the equipment. Thus, to determine optical retardation (Δn) of the samples in nm, the angle (α) read in the microscope is multiplied by 3.03 nm [32]. Five α readings were performed for each histological section.

### Statistical analysis

Shapiro-Wilk test revealed that the variables did not follow Gaussian (normal) distribution, except for the myofibroblast count. Thus, the Mann-Whitney test was used to detect differences between all possible pairs in the within-day analyses for all variables except the myofibroblast count, for which the Student’s t-test was used. The Minitab 16 software program (Minitab Inc, USA) was used for all statistical analyses, with the level of significance set to 95% (α = 0.05).

## Results and Discussion

**Fig 1:**
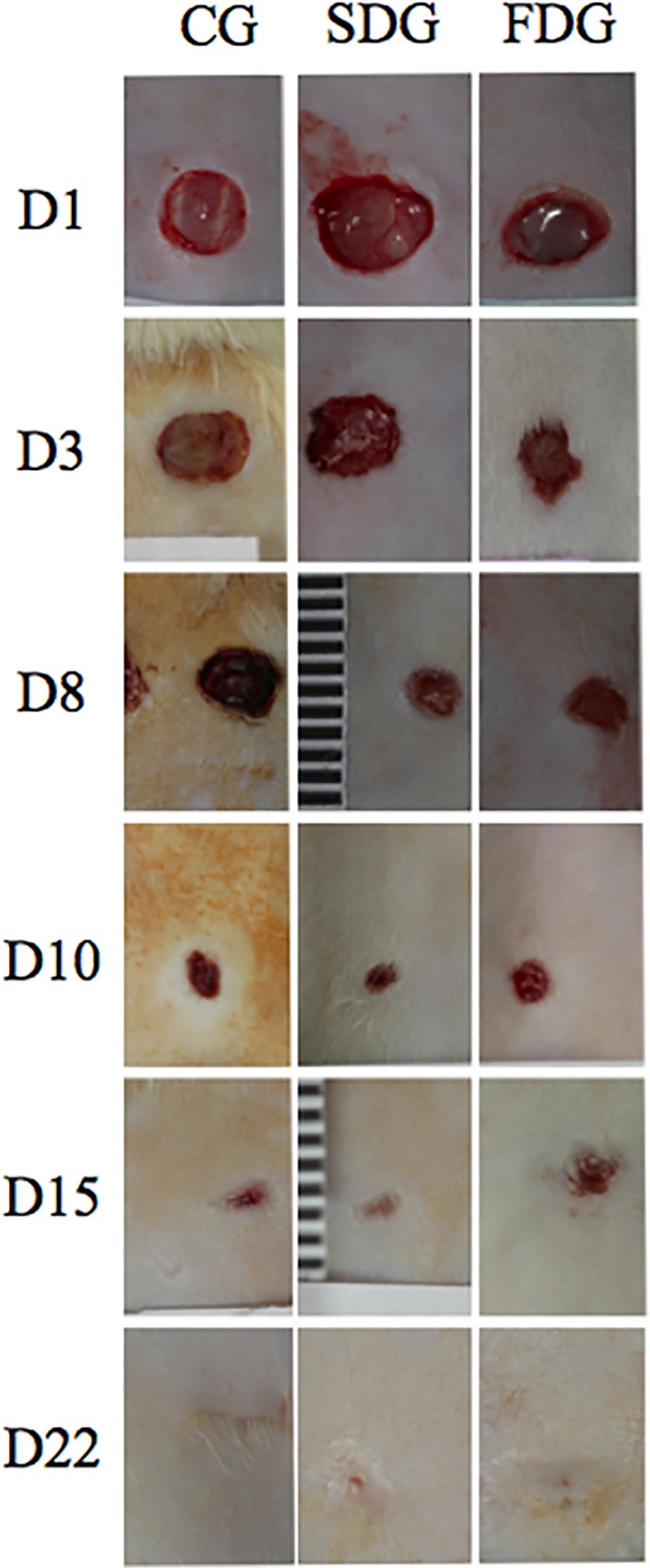
Wound healing in different groups throughout experiment (D = Day, CG = Control Group, SDG = Single Dose Group, FDG = Fractionated Dose Group). Original magnification 10x)

**Fig 2:**
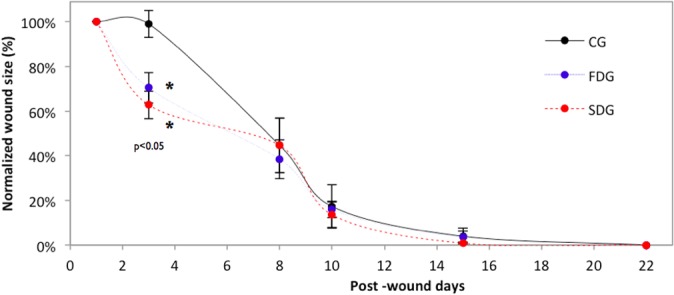
Percentage of normalized wound closure throughout experiment demonstrating the effect of laser therapy in the early tissue repair process. (mean ± SEM, *p*<0.05)

The present findings demonstrate that laser therapy delivered either once or four times enhances the early phase of tissue repair by accelerating initial wound closure and leukocyte chemotaxis, with more myofibroblasts and more organized fibrous tissue in the wound. Independently of the energy density, laser therapy accelerated wound closure by approximately 40% in the first three days in comparison to the control group, after which the closure rate decreased in a similar rate in all groups from Day 8 onward (Figs 1 and 2).

On Day 3, the laser groups had a significantly smaller injury area in comparison to the control group. Beginning with Day 8, no statistically significant differences in injury area were found among the groups, and the ulcer was no longer apparent in any group by Day 22 (Fig 2).

Thus, it cannot be stated that laser therapy accelerated the healing process, as wound closure did not occur earlier in any group in comparison to the other groups, which is in agreement with data reported in previous studies [33, 34]. However, the biomodulation caused by laser therapy was sufficient to achieve perceptible effects in the inflammatory phase of the healing process (Day 3), which can be of considerable assistance in major surgeries to which patients with diabetes are submitted, exposing such patients to a lower risk of infection.

**Fig 3:**
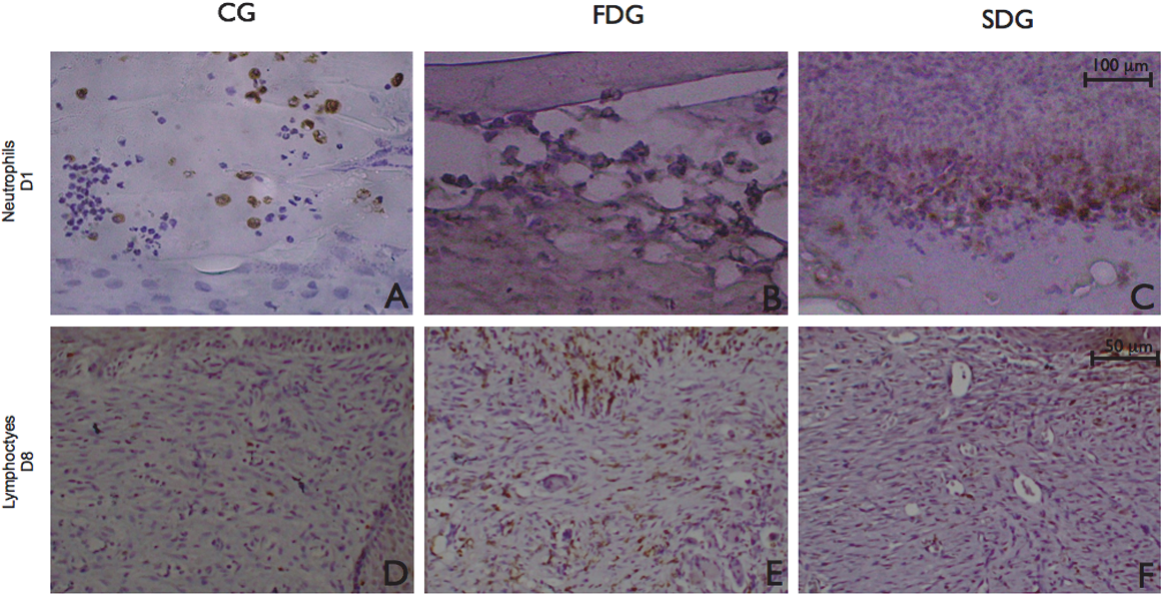
Histological examination of healing tissue—Day 3: all groups in inflammatory phase of tissue repair (A, B, C), with a crust over the ulcer and an intense inflammatory infiltrate (*); Day 8: wounds in proliferative phase with granulation tissue (D, E, F); Some samples in FDG exhibited acute inflammatory infiltrate at this time (*). hematoxilyn & eosin staining; original magnification: 200 x

**Fig 4:**
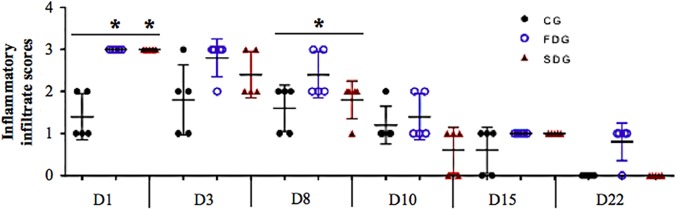
Inflammatory infiltrate score—SDG exhibited more leukocytes than other groups through to Day 10.

Laser therapy increased acute inflammatory infiltrate measured by neutrophils count two hours after the induction of the wound, which remained high through to Day 3. From Day 8 onward, acute inflammatory infiltrate was gradually replaced with chronic infiltrate, measured by the T lymphocyte count, with significant differences among the groups (Figs 3 and 4).

In patients with diabetes, the inflammatory response following injury is often prolonged and excessive [15] due to dysregulated coagulation and inflammatory response [35] as well as protein glycation, which leads to thicker capillary basal membranes with altered permeability. The migration of inflammatory cells to and from the injury site is delayed, resulting in chronic inflammation. Studies report that low-level laser therapy increases neutrophil chemotaxis [18, 36]. In the present investigation, laser therapy triggered leukocyte chemotaxis (especially neutrophils) beginning at two hours after injury, with a peak on Day 3, independently of the energy density (1 or 4 J/cm^2^) (Fig 4). It should be stressed that the group submitted to a single-dose of low-level laser irradiation had the best inflammatory response, as demonstrated by the significantly higher scores from Day 3 to Day 10 in comparison to the other groups.

**Fig 5:**
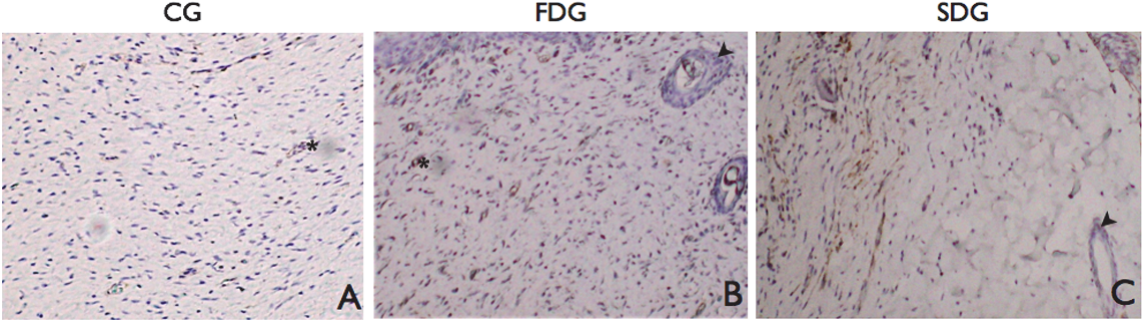
Immunohistochemical analysis with anti-α smooth muscle actin; myofibroblasts within granulation tissue stained brown; arrow heads point to smooth muscle in hair follicles; asterisks indicate smooth muscle in arterioles used as internal positive control of reaction; immunohistochemistry with DAB; original magnification: 400 x

Laser application altered the inflammatory infiltrate trend line (Fig 5). Independently of the energy density (1 or 4 J/cm^2^), laser therapy induced leukocyte chemotaxis in the early stages of tissue repair. The leukocytes scores were similar among the different groups from Day 8 onward.

As expected, myofibroblasts appeared on Day 8 and remained through to Day 22 in all groups (Fig 5).

On Day 15, the number of myofibroblasts began to decrease in the control group, but continued to increase in the laser groups. On Day 22, all groups had fewer myofibroblasts, demonstrating that the scars were in the remodeling phase, but both laser groups had more of these cells than the control group.

**Fig 6:**
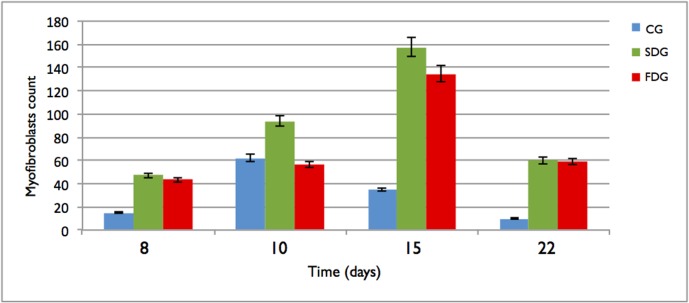
Myofibroblast count showing more cells in both laser groups on Days 8, 15 and 22 in comparison to control; SDG exhibited more myofibroblasts than other groups on Days 10 and 15. Bars represent mean counts with respective standard deviation values.

The proliferative phase of tissue repair is characterized by the formation of fibrous tissue and angiogenesis and is strongly modulated by transforming growth factor beta (TGF-β), which induces the proliferation of fibroblasts and their differentiation into myofibroblasts. Laser induces the appearance of myofibroblasts in granulation tissue during the proliferation and remodeling phases of the tissue repair process, likely through the modulation of TGF-β synthesis. Szymanska et al. (2013) demonstrated that LLLT at a wavelength of 635 nm increases endothelial cell proliferation, with a corresponding decrease in the concentration of vascular endothelial growth factor, suggesting the role of this growth factor in this process; in contrast, the 830 nm wavelength was associated with a decrease in TGF-β secretion [22]. Visible red laser (660 nm) was employed in the present study and led to a significant increase in the appearance of myofibroblasts in the healing process, especially on Days 15 and 22 (Fig 6), which may be helpful to wound contraction and remodeling in patients with diabetes.

**Fig 7:**
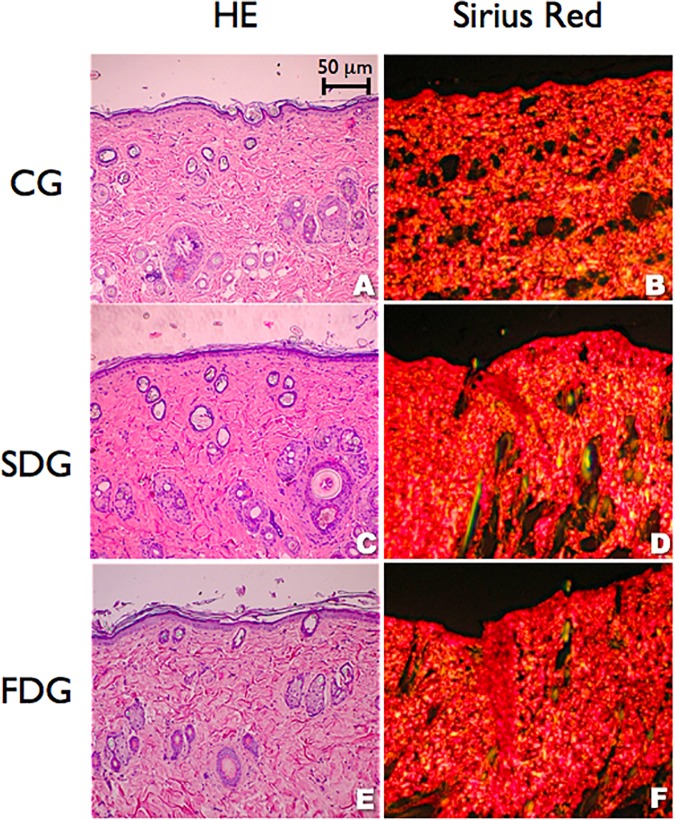
Morphology of wound healing on Day 22, showing similarity among experimental groups (hematoxilyn and eosin—A, C, E; Picrosirius Red—B D, F)

Interference colors with the use of Picrosirius Red and polarized light revealed that laser therapy did not affect the proportion of larger, more packed collagen fibers in relation to thinner, less packed fibers on Day 22 (Fig 7). However, the optical retardation analysis demonstrated that the collagen fibers were significantly more organized in the SDG. All wounds in the three groups were closed by Day 22, with well-formed epithelium showing a mature stratum corneum and epidermal appendages (sebaceous glands and hair follicles). The skin barrier function is mainly assigned to the stratum corneum layer of the epidermis, which prevents exogenous substances from entering the body while also minimizing transepidermal water loss.

**Fig 8:**
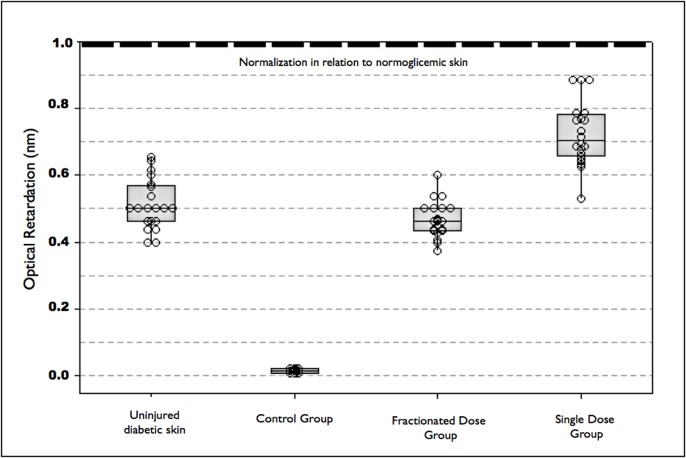
Optical retardation analysis of collagen on Day 22, showing most organized collagen in SDG, followed by uninjured skin, FDG and CG

On Day 22, the collagen fibers were more organized in the SDG in comparison to intact, uninjured diabetic skin. The FDG had slightly less organized tissue than uninjured skin and the control group exhibited the worst degree of collagen organization (Fig 8).

Using second harmonic generation (SHG), which is sensitive to the molecular orientation of collagen fibers, Kim et al. found that changes in protein structure caused by glycation lead to a reduction in the signal measured by SHG, indicating less organization of the fibers [37]. In the present study, optical retardation was employed for the inference of the molecular orientation of the collagen fibers, as this method is well established, more accessible and correlated with the signal obtained through SHG [32]. Since tissue glycation is a biochemical characteristic of diabetes, a low degree of molecular organization was expected and, consequently, low optical retardation. However, the SDG exhibited significantly different values in comparison to the other groups, including in comparison to uninjured diabetic tissue. The buildup of glycation products can lead to the loss of organization in the fibrillar arrangement, which is reflected in the fibers, bundles and extracellular matrix [38]. As both laser groups had significantly greater optical retardation in comparison to the control group, one may infer that the laser-glycated tissue interaction is an important factor in the organization of collagen fibers in individuals with diabetes. Moreover, the energy density employed should be considered in this interaction, as greater collagen organization was found in the SDG than the FDG.

Diabetic wounds fail to form adequate granulation tissue; angiogenesis is poor and the scar does not contract properly, often resulting in dehiscence or chronic wounds. No previous studies in the literature report an increase in collagen synthesis and deposition following laser therapy [20]. In the present investigation, however, collagen organization was significantly affected by laser therapy as well as the energy density. A single laser application of 4 J/cm^2^ after surgery led to better collagen organization in the scar tissue, demonstrating that the initial inflammatory events following an injury are crucial to the final modulation of the repair process. The fractionated dose (four applications of 1 J/cm^2^) also led to improved final scar quality, but the energy density in the inflammatory phase may not have been sufficient to accelerate this phase of the tissue repair process in rats with diabetes. However, cells and tissues under stress are more prone to be influenced by laser biomodulation and low fractionated doses are believed to be better than a single dose for “normally responding” tissue [16].

## Conclusion

The present findings demonstrate that low-level laser therapy in the immediate postoperative period can enhance the tissue repair process in patients with diabetes by modulating the inflammatory process, increasing the synthesis of myofibroblasts and enhancing collagen organization. Similar effects were achieved with laser therapy applied a single time with an energy density of 4 J/cm^2^ and applied four times with an energy density of 1 J/cm^2^. Moreover, the application of laser therapy in the inflammatory phase was the most important factor to the enhancement of the tissue repair process. Further studies should be conducted to determine the role of the red wavelength on the modulation of glycation through biochemical analysis and compare the advantages of different energy delivery regimens (single higher dose or multiple lower doses).
